# The origins and dynamic changes of C3- and S100A10-positive reactive astrocytes after spinal cord injury

**DOI:** 10.3389/fncel.2023.1276506

**Published:** 2023-12-22

**Authors:** Qing Zhao, Yi-long Ren, Yan-jing Zhu, Rui-qi Huang, Rong-rong Zhu, Li-ming Cheng, Ning Xie

**Affiliations:** ^1^Key Laboratory of Spine and Spinal Cord Injury Repair and Regeneration of Ministry of Education, Orthopaedic Department of Tongji Hospital, School of Medicine, School of Life Sciences and Technology, Tongji University, Shanghai, China; ^2^Division of Spine, Department of Orthopedics, Tongji Hospital, Tongji University School of Medicine, Tongji University, Shanghai, China; ^3^Clinical Center for Brain and Spinal Cord Research, Tongji University, Shanghai, China

**Keywords:** spinal cord injury, C3- and S100A10-positive reactive astrocytes, origins, distribution, dynamic changes

## Abstract

Accaumulating studies focus on the effects of C3-positive A1-like phenotypes and S100A10-positive A2-like phenotypes of reactive astrocytes on spinal cord injury (SCI), however the origins and dynamic changes of C3- and S100A10-positive reactive astrocytes after SCI remain poorly understood. Through transgenic mice and lineage tracing, we aimed to determine the origins of C3- and S100A10-positive reactive astrocytes. Meanwhile, the distribution and dynamic changes in C3- and S100A10-positive reactive astrocytes were also detected in juvenile and adult SCI mice models and cultured astrocytes. Combing with bulk RNA sequencing (RNA-seq), single-cell RNA sequencing (scRNA-seq) and bioinformatic analysis, we further explored the dynamic transcripts changes of C3- and S100A10-positive reactive astrocytes after SCI. We confirmed that resident astrocytes produced both C3- and S100A10-positive reactive astrocytes, whereas ependymal cells regenerated only S100A10-positive reactive astrocytes in lesion area. Importantly, C3-positive reactive astrocytes were predominantly activated in adult SCI mice, while S100A10-positive reactive astrocytes were hyperactivated in juvenile mice. Furthermore, we observed that C3- and S100A10-positive reactive astrocytes had a dynamic transformation process at different time *in vitro* and *vivo*, and a majority of intermediate states of C3- and S100A10-positive reactive astrocytes were found during transformation. RNA-seq and scRNA-seq results further confirmed that the transcripts of C3-positive reactive astrocytes and their lipid toxicity were gradually increased with time and age. In contrast, S100A10-positive reactive astrocytes transcripts increased at early time and then gradually decreased after SCI. Our results provide insight into the origins and dynamic changes of C3- and S100A10-positive reactive astrocytes after SCI, which would be valuable resources to further target C3- and S100A10-positive reactive astrocytes after SCI.

## 1 Introduction

Astrocytes, as the most abundant cells in the central nervous system, provide trophic support to neurons and regulate synapse formation and homeostasis (Sofroniew and Vinters, 2010). Astrocytes strongly activate after spinal cord injury (SCI) and proliferate to form reactive astrocytes and dense glial scars as a physical barrier to axonal regeneration. A generally accepted view is that astrocytes have dual functions in inhibiting and promoting axon regeneration (Sabelström et al., 2013; Anderson et al., 2016; Gong et al., 2022). The recently defined C3- and S100A10-positive reactive astrocytes may be an important reason for this phenomenon (Liddelow et al., 2017). Complement C3 and S100 family protein p11 (S100A10) were identified and widely used as characteristic markers of C3-positive reactive astrocytes and S100A10-positive reactive astrocytes, respectively (Liddelow et al., 2017). Meanwhile, increasing studies have focused on the role of C3- and S100A10-positive reactive astrocytes in neural repair after SCI (Vismara et al., 2020; Li et al., 2021; Liu et al., 2023; Wang et al., 2023).

Previous studies showed that inhibiting NF-κB P65 phosphorylation could reduce C3-positive reactive astrocytes activation and neuronal apoptosis, and promote axon regeneration and functional recovery after SCI (Wang et al., 2018; Liu et al., 2019). Intriguingly, a number of studies have shown that C3-positive reactive astrocytes can transform into S100A10-positive reactive astrocytes (Li T. et al., 2020; Wang et al., 2021; Liu et al., 2022). However, there are still some unresolved issues. Especially, a recent study uncovered six astrocyte subtypes at single-cell resolution with new signatures during the long-term pathological process after SCI (Hou et al., 2022). Nonetheless, the origins and dynamic changes of C3- and S100A10-positive reactive astrocytes after SCI remain poorly understood. In this study, we investigated the origins of C3- and S100A10-positive reactive astrocytes through using transgenic mice and lineage tracing after SCI. By constructing SCI models of juvenile and adult mice, we tried to observe the distribution and dynamic changes of C3- and S100A10-positive reactive astrocytes. Combining RNA sequencing (RNA-seq), single-cell RNA sequencing (scRNA-seq) and bioinformatic analysis, we further attempted to detect the dynamic transcripts changes of C3- and S100A10-positive reactive astrocytes. We also detected their dynamic changes and transformation process *in vitro* under inflammatory and hypoxic condition.

## 2 Materials and methods

### 2.1 Experimental models (organisms/strains) and SCI models

All procedures were carried out in accordance with protocols approved by the Institutional Animal Care and Use Committee (IACUC) of the Tongji University School of Medicine. Mice were housed on a 12/12-h light/dark cycle and had food and water available *ad libitum*. The room temperature and humidity were appropriate. C57BL/6-Foxj1^*em*1(*GFP*−*CreERT*2−*polyA*)*Smoc*^ mice (Cat# NM-KI-200133) were obtained from the Model Organisms Center (Shanghai, China). B6.Cg-*Gt(ROSA) 26Sor* ^*tm*9(*CAG*−*tdTomato*)*Hze*^/^J^ mice (Cat# JAX007909) were obtained from the Jackson Laboratory. The transgenic mice and genotyping primers are listed in Supplementary Table 1.

C57BL/6-Foxj1^*em*1(*GFP*−*CreERT*2−*polyA*)*Smoc*^ female mice (*n* = 3) and 8-week-old wild-type female mice (*n* = 3, Shanghai Jiesijie Laboratory Animal Co., Ltd.) were used for spinal cord contusion models. B6.Cg-*Gt(ROSA) 26Sor* ^*tm*9(*CAG*−*tdTomato*)*Hze*^/^J^ female mice (*n* = 3), 8-week-old wild-type female mice (sham group, *n* = 9; SCI group, *n* = 9), 1-week-old (sham group, *n* = 6; SCI group, *n* = 6) and 2-week-old juvenile mice (sham group, *n* = 6; SCI group, *n* = 6), were used for spinal cord crush model construction. Contusion and crush models at T10 were established as we previously reported (Zhao et al., 2022). The sham-treated mice received laminectomy without contusion and crush. Mice had received natural illumination to keep warm before and after the surgery. Juvenile mice were returned to the female mice cage to continue feeding after surgery. Bladders of the injured mice were manually expressed twice per day until autonomous urination recovered.

### 2.2 AAV2/5-GfaABC1D-Cre injection

AAV2/5-GfaABC1D-Cre has been identified as the most reliable virus and is widely used to selectively target astrocytes (Cui et al., 2018; Nagai et al., 2019). AAV2/5-GfaABC1D-Cre was obtained from Shanghai Taitool Bioscience Co., Ltd. (Cat# S0611-5, ≥1E+13 V.G/ml). B6.Cg-*Gt(ROSA) 26Sor* ^*tm*9(*CAG*−*tdTomato*)*Hze*^/^J^ mice were used for AAV2/5-GfaABC1D-Cre injection at 2 weeks before injury. A laminectomy was performed at T10 as described above. AAV2/5-GfaABC1D-Cre was injected into the both sides of dorsal median of T10 (0.5 mm lateral to midline with a depth of 0.6 mm, 0.4 μL per side, ≥0.33E+11 V.G/ml in sterile saline), with a Hamilton 33-gauge (33 G) microinjection needle at a speed of 0.2 μL/min. After injection, the needle was held for 5 min to avoid liquid reflux.

### 2.3 Immunofluorescence staining and quantitative image analysis

The preparation of tissue slices, immunofluorescence experiments and analysis were carried out based on our previous methods (Zhao et al., 2022). Mice were euthanized at 2 weeks post injury (2 WPI). The primary antibodies were as follows: glial fibrillary acidic protein (GFAP, Cat# ab4674, Abcam, 1:500), S100 family protein p11 (S100A10, Cat# 11250-1-AP, Protein Tech, 1:250), and complement C3 (C3, Cat# sc-528410, Santa Cruz Biotechnology, 1:50); the secondary antibodies were Alexa^®^ Fluor 488 (Cat# abs20019A, Absin, 1:500), Alexa^®^ Fluor 488 (Cat# A-32814, Invitrogen, 1:500), Alexa^®^ Fluor 555 (Cat# A-32773, Invitrogen, 1:500), Alexa Fluor^®^ 594 (Cat# ab150156, Abcam, 1:500), Alexa^®^ Fluor 594 (Cat# ab150176, Abcam, 1:500), and Alexa^®^ Fluor 647 (Cat# A-32795, Invitrogen, 1:500). Fluorescent antibodies of the same channel including Alexa^®^ Fluor 488 and Alexa^®^ Fluor 594, were used to match species differences during immunofluorescence staining.

### 2.4 RNA sequencing and bioinformatic analysis

To further reveal the dynamic changes in C3- and S100A10-positive reactive astrocytes, we used RNA-seq to examine the transcriptomes of the lesion site at 2 weeks post injury (2 WPI), including sham and SCI group of 1-week-old mice (sham_1W, *n* = 3; SCI_1W, *n* = 3); sham and SCI group of 2-week-old mice (sham_2W, *n* = 3; SCI_2W, *n* = 3); sham and SCI group of 8-week-old mice (sham_8W, *n* = 3; SCI_8W, *n* = 3). RNA-seq was carried out as we previously described (Zhao et al., 2022). The sequence and sample data have been deposited in NCBI database under Sequence Read Archive (SRA) with Bioproject identification number PRJNA847738 (Accession number: SRR19612226 - SRR19612243). Bioinformatics analysis, including heatmap, Gene Ontology (GO) enrichment and Kyoto Encyclopedia of Genes and Genomes (KEGG) pathway was carried out with the online platform Dr. Tom (BGI Company). Differentially expressed genes (DEGs) were identified using the DEGseq2 method and screened with the criteria of *q*-value ≤ 0.05 and log2FC ≥ 0.6 (fold change, FC). The scRNA-seq results were analyzed through online data from injured mouse spinal cords (https://jaeleelab.shinyapps.io/sci_singlecell/) (Milich et al., 2021). The series matrix file data of GSE45006 were downloaded from the Gene Expression Omnibus (GEO) public database, and the annotation platform was GPL1335 as we previously reported (Zhao et al., 2022). DEGs were identified using the DEGseq method and screened with the criteria of *P*-value ≤ 0.01 and log2FC ≥ 2. Statistical analyses were conducted in R language (version 4.1). The R code was modified from https://github.com/jmzeng1314/GEO.

### 2.5 Astrocyte culture

Primary astrocyte cultures were obtained from the cerebral cortices of postnatal day 1–2 (P1–2) Sprague Dawley rats (Shanghai Jiesijie Laboratory Animal Co., Ltd.). Briefly, the cerebral cortices were digested by papain (5 mg/ml, Cat# LS003126, Worthington) and seeded on 0.01% poly-D-lysine-coated (Cat# C0312, Beyotime Institute of Biotechnology, China) 75-cm^2^ flasks. Dissociated cells were maintained in DMEM-F12 (Cat# 11330057, Gibco) containing 10% fetal bovine serum (FBS, Cat# 16000-044, Gibco) and 1% penicillin/streptomycin at 37°C for 7 days. Adherent cells were dissociated by Accutase (Cat# A1110501, Gibco) after reaching 70–80% confluency for 10 min at 37°C and then reseeded in 100 mm culture dishes (430167, Corning) for 1 h. Then, the supernatant fluid was discarded to remove microglia and oligodendrocytes. Astrocytes with > 95% purity were obtained and reseeded in poly-D-lysine-coated 12-well culture plates with cell climbing sheets for next experiment. To mimic hypoxic conditions to induce S100A10-positive reactive astrocytes as previously reported (Zamanian et al., 2012), we generated the oxygen glucose deprivation (OGD, O_2_ < 0.1%) model with an AnaeroPack (Cat# D07, Mitsubishi Gas Chemical Company, Japan) in a 2.5 L closed plastic box (Zhao et al., 2022). After reaching 70–80% confluency, astrocytes were incubated under hypoxic conditions with DMEM-no glucose (Cat# 11966025, Gibco) for 3 h and then switched to DMEM-F12 containing 10% FBS and 1% penicillin/streptomycin for 8, 24, and 72 h. Then, astrocytes were collected at different times for quantitative real-time PCR (RT-PCR) and immunofluorescence staining as we described previously (Zhao et al., 2022). OGD control wells were established, and the data were analyzed and compared with those of the control wells. Image-J software with customized macros was used to quantify the cell area and diameter of astrocytes.

### 2.6 Microglial culture

To obtain C3-positive reactive astrocytes as previously reported (Liddelow et al., 2017), we used activated microglia induced by lipopolysaccharides (LPS, Cat# Escherichia coli O111:B4, Sigma-Aldrich) in this study. Mouse microglial SIM-A9 cells were purchased from ATCC (Cat# CRL-3265, ATCC) and cultured according to ATCC recommended growth conditions in DMEM-F12 (Cat# 11330057, Gibco) containing 10% heat-inactivated FBS (Cat# 16140-071, Gibco) and 5% heat-inactivated horse serum (Cat# 26050-088, Gibco) (Correia et al., 2021). LPS at concentrations of 2 and 0.2 μg/mL LPS was used to stimulate SIM-A9 cells after they reached 60–70% confluency for 24 h. Microglia culture medium was collected and centrifuged at 1,200 × g for 5 min to remove debris. The expression of pro-inflammatory and anti-inflammatory microglial marker genes were measured by RT-PCR. The conditioned media of microglia stimulated with 2 and 0.2 μg/mL LPS were transferred to and activated astrocytes in 12-well culture plates for 8, 24, and 72 h. Then, astrocytes were collected for RT-PCR and immunofluorescence staining. LPS control wells were also established, and the data were analyzed and compared with those of the control wells.

### 2.7 Quantitative real-time PCR

RT-PCR was performed as we previously reported (Zhao et al., 2022). The primer sequences were designed as previously reported (Wang et al., 2021) or through PrimerBank and were listed in Supplementary Table 2. The relative mRNA levels were calculated using the ΔΔCt relative quantification method as our previous reported (Zhao et al., 2022). GAPDH served as the control gene, and the mRNA levels of specific genes were normalized to that of GAPDH. The group without both OGD and conditioned media of microglia with LPS stimulation was used as the control group, and RNA was also isolated at the same time points as OGD and LPS stimulation. The data were analyzed and compared with control group. Calculations and statistical analyses were performed in Microsoft Excel version 16.36. Graphs were plotted in GraphPad Prism 8 version 8.4.3.

### 2.8 Statistical analysis

All experiments were conducted with three or more duplicates. All continuous data were shown as mean ± SEM. One-way ANOVA was performed followed by Student Newman–Keuls *post-hoc* test for continuous data. The method of two-way ANOVA was used for age-dependent data. *P*-values < 0.05 were considered statistically significant. Data analyses were conducted using the Statistical Analysis System (SAS), version 9.4 (SAS Institute, Inc, Cary, NC, USA). Plots were generated using GraphPad Prism 8 software (GraphPad Software, San Diego, CA).

## 3 Results

### 3.1 C3- and S100A10-positive reactive astrocytes had different lineage origins

**Figure 1:**
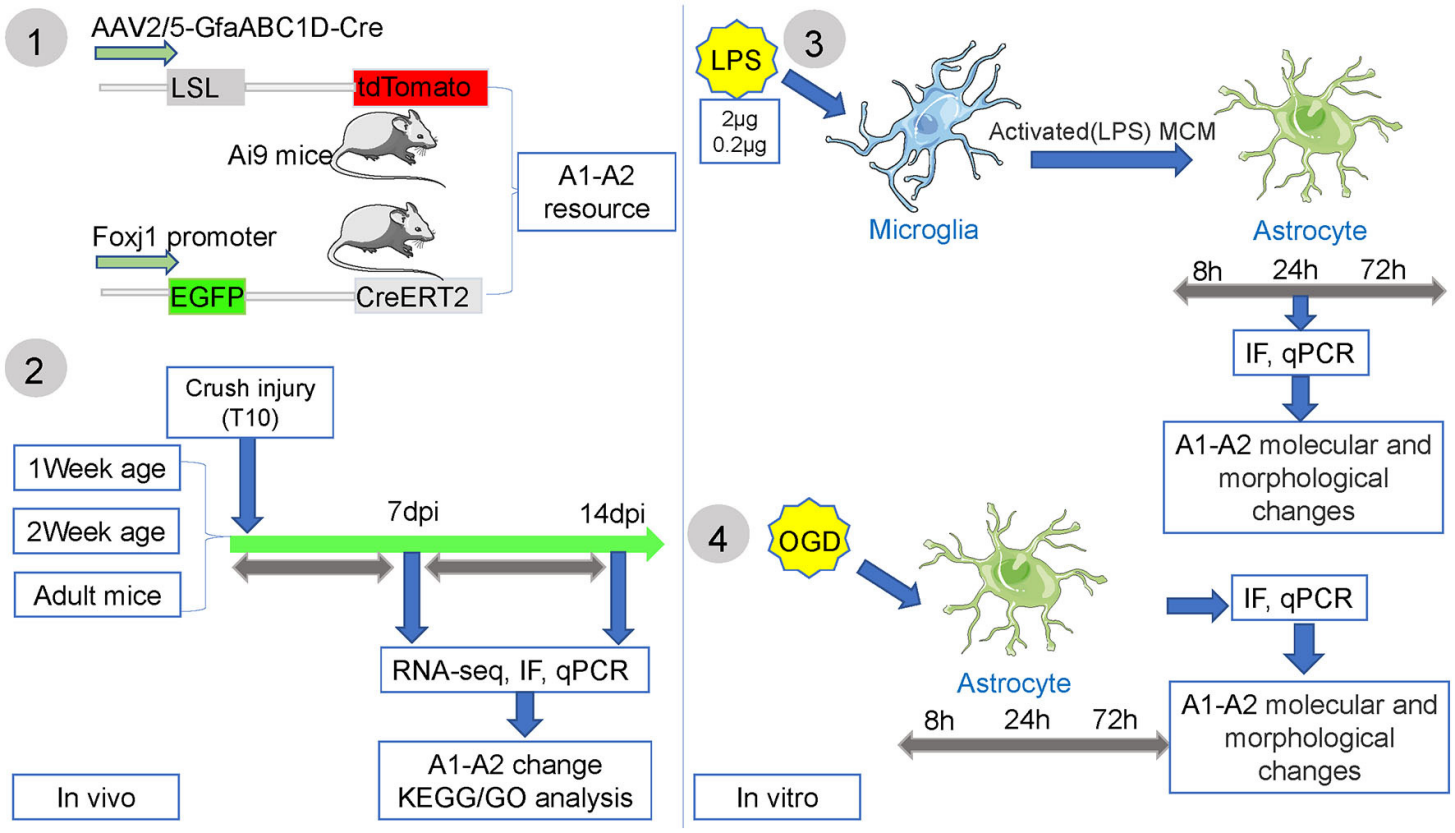
The experimental flow chart of this study.

**Figure 2:**
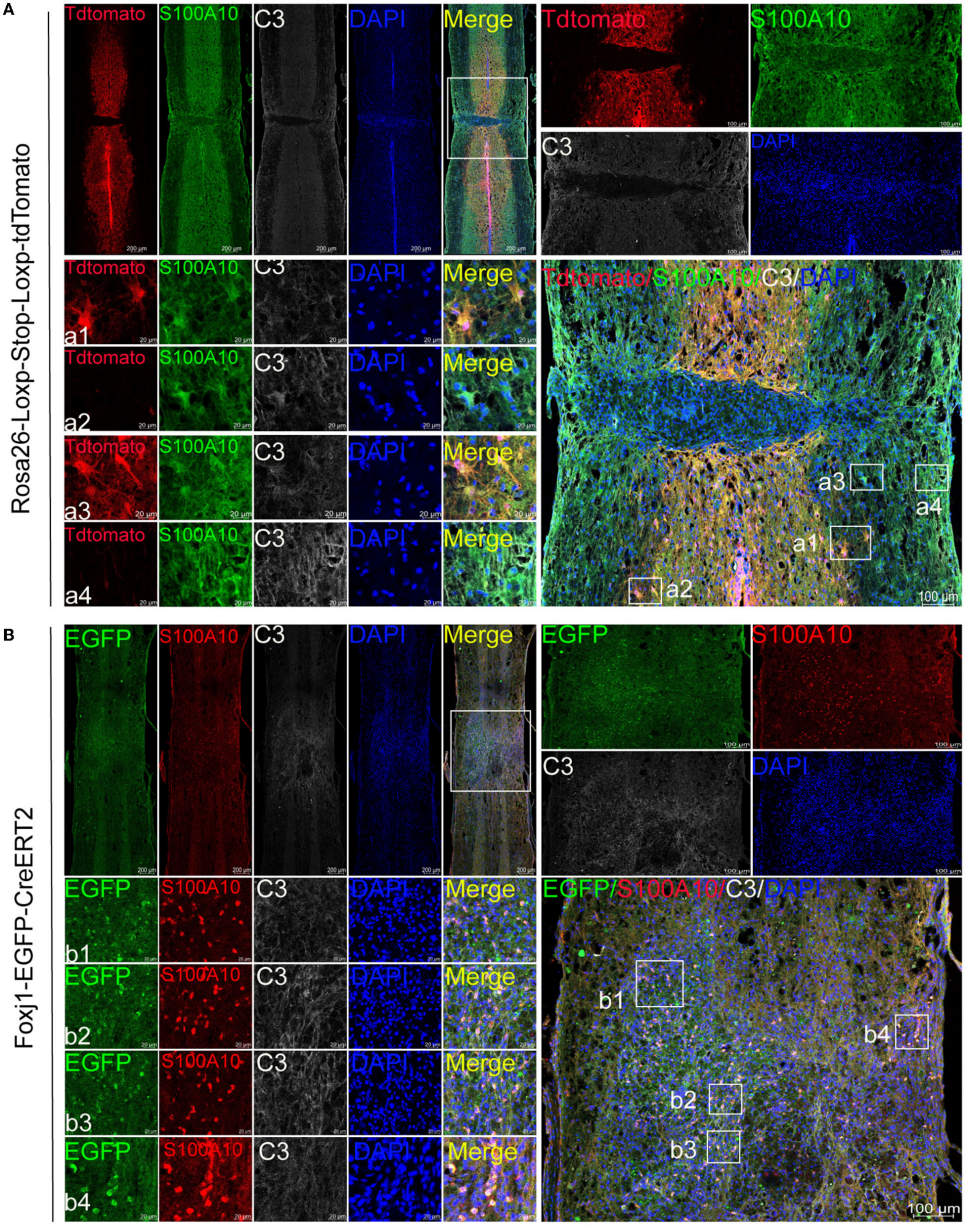
C3- and S100A10-positive reactive astrocytes had different lineage sources. **(A)** AAV2/5-GfaABC1D-Cre was used to specifically label resident astrocytes in B6.Cg-*Gt(ROSA) 26Sor* ^*tm*9(*CAG*−*tdTomato*)*Hze*^/^J^ mice. Images of immunofluorescent staining using C3 (white) and S100A10 (green) as characteristic markers of C3- and S100A10-positive reactive astrocytes, respectively. TdTomato indicated tdTomato+ astrocytes in Rosa26-Loxp-Stop-Loxp-tdTomato mice. *n* = 3 biological repeats. Scale bars are indicated in the pictures. **(B)** C57BL/6-Foxj1^*em*1(*GFP*−*CreERT*2−*polyA*)*Smoc*^ transgenic mice were used for ependymal cells lineage tracing. S100A10 (red), S100A10-positive reactive astrocytes marker. EGFP indicated EGFP+ ependymal cells in C57BL/6-Foxj1^*em*1(*GFP*−*CreERT*2−*polyA*)*Smoc*^ mice. *n* = 3 biological repeats. Scale bars are indicated in the pictures.

The experiment was carried out as shown in Figure 1. Immunofluorescence results showed that astrocytes (tdTomato+) were labeled by AAV2/5-GfaABC1D-Cre in the lesion site and edge after SCI (Figure 2A). We found that resident astrocytes could generate both C3- and S100A10-positive reactive astrocytes (Figure 2A). In particular, a number of tdTomato+ C3+ S100A10+ triple-positive cells were found after SCI (Figure 2A). Recent studies have shown that C3- and S100A10-positive reactive astrocytes could transform into each other (Li T. et al., 2020; Wang et al., 2021), and we speculated that these cells may be the intermediate state of C3- and S100A10-positive reactive astrocytes. Ependymal cells also give rise to a substantial proportion of scar-forming astrocytes after SCI (Meletis et al., 2008). To target ependymal cells in the spinal cord, we used C57BL/6-Foxj1^*em*1(*GFP*−*CreERT*2−*polyA*)*Smoc*^ mice with the reporter gene EGFP to establish a contusion SCI model and examined the activation and differentiation of ependymal cells. The slices from the SCI epicenter showed that ependymal cells produced a large number of S100A10-positive cells in the epicenter but not the edge (Figure 2B). Therefore, ependymal cells may be involved in the production of S100A10-positive reactive astrocytes. In total, resident astrocytes produced both C3- and S100A10-positive reactive astrocytes after SCI, while ependymal cells regenerated only S100A10-positive reactive astrocytes in our transgenic mice.

### 3.2 The distribution of C3- and S100A10-positive reactive astrocytes was different between juvenile and adult mice after SCI

**Figure 3:**
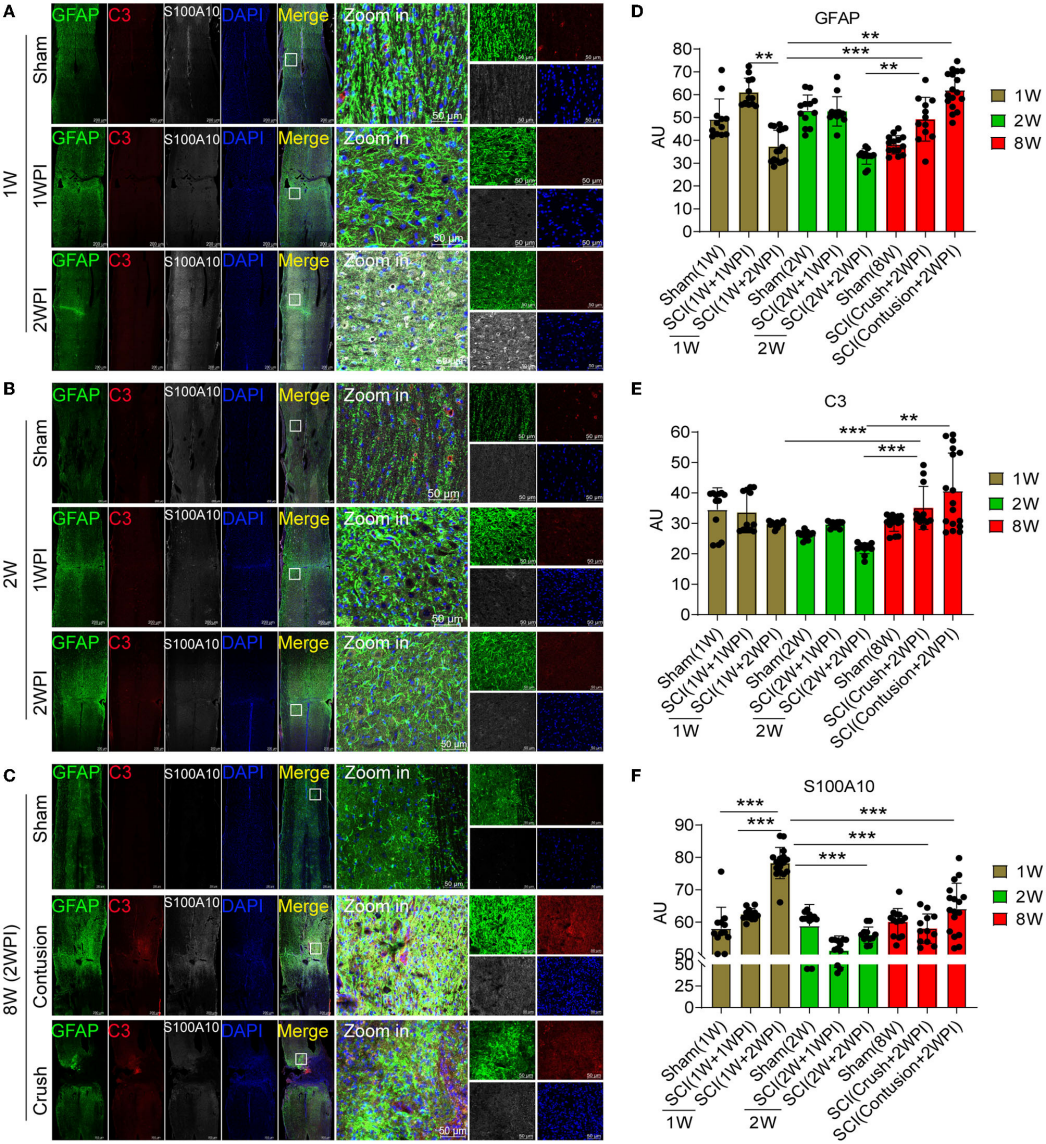
The distribution of C3- and S100A10-positive reactive astrocytes was significantly different between the juvenile and adult mice after SCI. **(A–C)** Images of immunofluorescent staining. Astrocyte marker GFAP (green), C3-positive reactive astrocytes marker C3 (red), S100A10-positive reactive astrocytes marker S100A10 (white). *n* = 3 biological repeats. Scale bars are indicated in the pictures. **(D–F)** Histogram of fluorescence intensity statistical results. *n* = 3 biological repeats. Values are the mean ± SEM. Statistical significance was determined by one-way ANOVA followed by Student Newman–Keuls *post-hoc* test. **P < 0.01, ***P < 0.001. 1 WPI, 1 week post injury; 2 WPI, 2 weeks post injury; 1 W, 1-week-old; 2 W, 2-week-old; 8 W, 8-week-old.

Juvenile mice have significant advantages in damage repair than adult mice after CNS injury, and this ability may be closely associated with astrocytes heterogeneity. Compared with those of the 8-week-old mice, astrocytes activation and glial scar formation of the juvenile mice (1- and 2-week-old) were not evident after SCI (Figures 3A–C). The motor function recovery of the juvenile mice was also better than that of the adult mice (data not shown). Compared with that in the sham group (Figure 3A), astrocytes activation (GFAP+) in 1-week-old mice was increased in the lesion area at 1 WPI (week post injury) and decreased at 2 WPI (Figures 3A, D). Compared with that of the 1-week-old (Figure 3A) and 2-week-old juvenile mice (Figure 3B) at 2 WPI, the activation of astrocytes was significantly increased in the 8-week-old mice (Figures 3C, D). There were no significant differences in C3-positive reactive astrocytes activation (C3+) between the 1- and 2-week-old groups (Figures 3A, B). C3-positive reactive astrocytes were rarely expressed in the sham groups and the SCI groups of 1- and 2-week-old juvenile mice (Figures 3A, B, E). Compared with that of the 1- and 2-week-old juvenile mice, C3-positive reactive astrocytes activation was significantly increased in the 8-week-old mice at 2 WPI (Figures 3C, E). S100A10-positive reactive astrocytes (S100A10+) were also rarely expressed at 1 WPI (Figures 3A–C). However, S100A10-positive reactive astrocytes activation in the 1-week-old mice was significantly increased at 2 WPI (Figures 3A, F) compared with that in the 2-week-old mice (Figures 3B, F) and the 8-week-old mice at 2 WPI (Figures 3C, F). In total, the activation of C3-positive reactive astrocytes were mainly activated in the 8-week-old mice, while the activation of S100A10-positive reactive astrocytes was significantly increased in the 1-week-old mice after SCI.

### 3.3 Bioinformatics analysis revealed the dynamic changes in C3- and S100A10-positive reactive astrocytes and lipid toxicity at different time points after SCI

**Figure 4:**
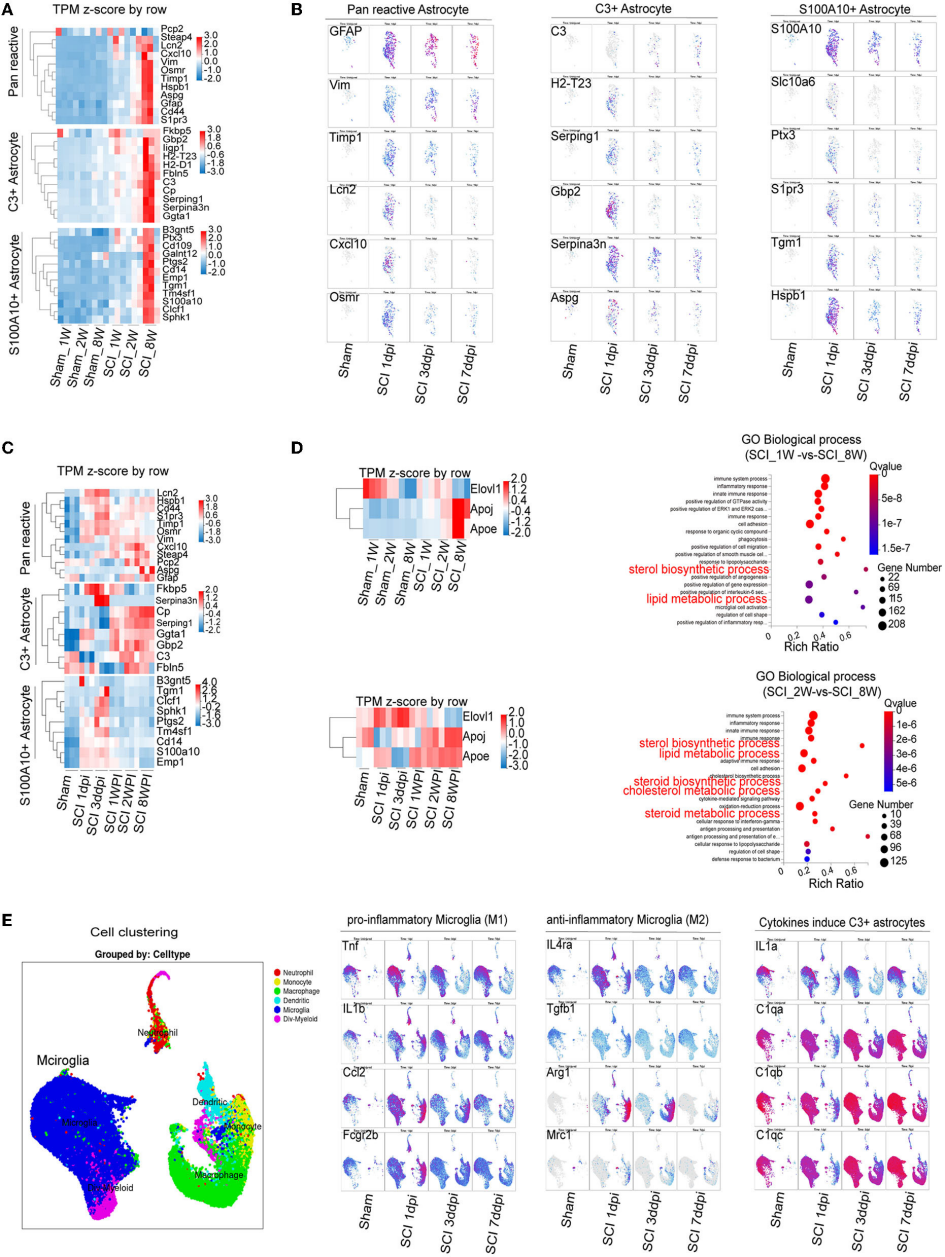
Combining RNA-seq and scRNA-seq data reveals the dynamic changes in C3- and S100A10-positive reactive astrocytes and lipid toxicity after SCI. **(A)** The heatmap showed elevation of C3- and S100A10-positive reactive astrocytes characteristic marker genes expression in different ages. *n* = 3 biological repeats. **(B)** scRNA-seq data from the injured mouse spinal cord revealed the dynamic changes in C3- and S100A10-positive reactive astrocytes at different time after SCI. **(C)** The heatmap reveals the dynamic changes in C3- and S100A10-positive reactive astrocytes at different time after SCI. *n* = 3 biological repeats. **(D)** The transcript of multiple lipid toxicity-related genes increased at different ages and time after SCI. The lipid metabolism-related pathways were gradually activated with age after SCI. *n* = 3 biological repeats. **(E)** The transcripts of pro-inflammatory microglia and the specific factors TNF-α/IL1/C1q inducing C3-positive reactive astrocytes were activated at different times after SCI. dpi, day post-injury; WPI, week post injury; 1 W, 1-week-old; 2 W, 2-week-old; 8 W, 8-week-old; sham_1 W, 1-week-old of sham group; sham_2 W, 2-week-old of sham group; sham_8 W, 8-week-old of sham group; SCI_1 W, 1-week-old of SCI group; SCI_2 W, 2-week-old of SCI group; SCI_8 W, 8-week-old of SCI group.

RNA-seq and bioinformatics analysis were used to reveal the dynamic changes of C3- and S100A10-positive reactive astrocytes after SCI between the juvenile and adult mice. However, when compared with the data of Figure 3, heatmap showed significant elevation of both C3- and S100A10-positive reactive astrocytes marker genes transcripts (Liddelow et al., 2017) in the SCI_8W group (Figure 4A). Through analyzing online scRNA-seq data from the injured mouse spinal cord (https://jaeleelab.shinyappsio/sci_singlecell/) (Milich et al., 2021), we found that pan-reactive astrocytes marker genes (*GFAP, VIM, Timp1, Lcn2, Cxcl10, Osmr*) were increased in astrocytes on the first day after SCI (Figure 4B). C3-positive reactive astrocytes (*C3, H2-T23, Serping1, Gbp2, Serpina3n, Aspg*) were also activated after SCI (Figure 4B). While, S100A10-positive reactive astrocytes (*S100A10, Slc10a16, PTX3, S1pr3, Tgm1, Hspb1*) were significantly activated on the first day after SCI and then gradually decreased (Figure 4B). By analyzing the RNA-seq data of the injured spinal cord at different time points, we found that astrocytes were gradually activated after SCI reflected by the pan-reactive astrocytes marker genes (Figure 4C). The heatmap revealed that C3-positive reactive astrocytes activation was not significant at the early stage after injury, gradually increased at 1 WPI and was sustained until 8 weeks (Figure 4C). Compared with C3-positive reactive astrocytes, S100A10-positive reactive astrocytes were significantly activated at the first day, peaked at the third day and then gradually decreased after injury (Figure 4C).

A recent study found that C3-positive reactive astrocytes caused axonal injury through saturated lipids contained in ApoE and APOJ lipid particles (Guttenplan et al., 2021). Lipotoxic ApoE/APOJ/Elovl1 was expressed in astrocytes at high levels after SCI (Gong et al., 2022). We also found that the transcripts levels of ApoE/APOJ/Elovl1 gradually increased with ages and time after SCI (Figures 4C, D). Lipid metabolism-related pathways were also activated (Figures 4C, D). Compared with those of anti-inflammatory microglia (*Il4ra, Tgfb1, Il10, Arg1, Mrc1*), the transcripts of pro-inflammatory microglial marker genes (*Tnf* , *Il1b, Il6, Ccl2, Fcgr2b*) were significantly increased after SCI (Figure 4E). Moreover, the specific factors TNF-α/IL1/C1q inducing C3-positive reactive astrocytes were obviously activated after SCI (Figure 4E), which could be the reason for C3-positive reactive astrocytes were gradually activated after SCI.

### 3.4 Dynamic changes and transformation process of C3- and S100A10-positive reactive astrocytes under inflammatory and hypoxic conditions *in vitro*

**Figure 5:**
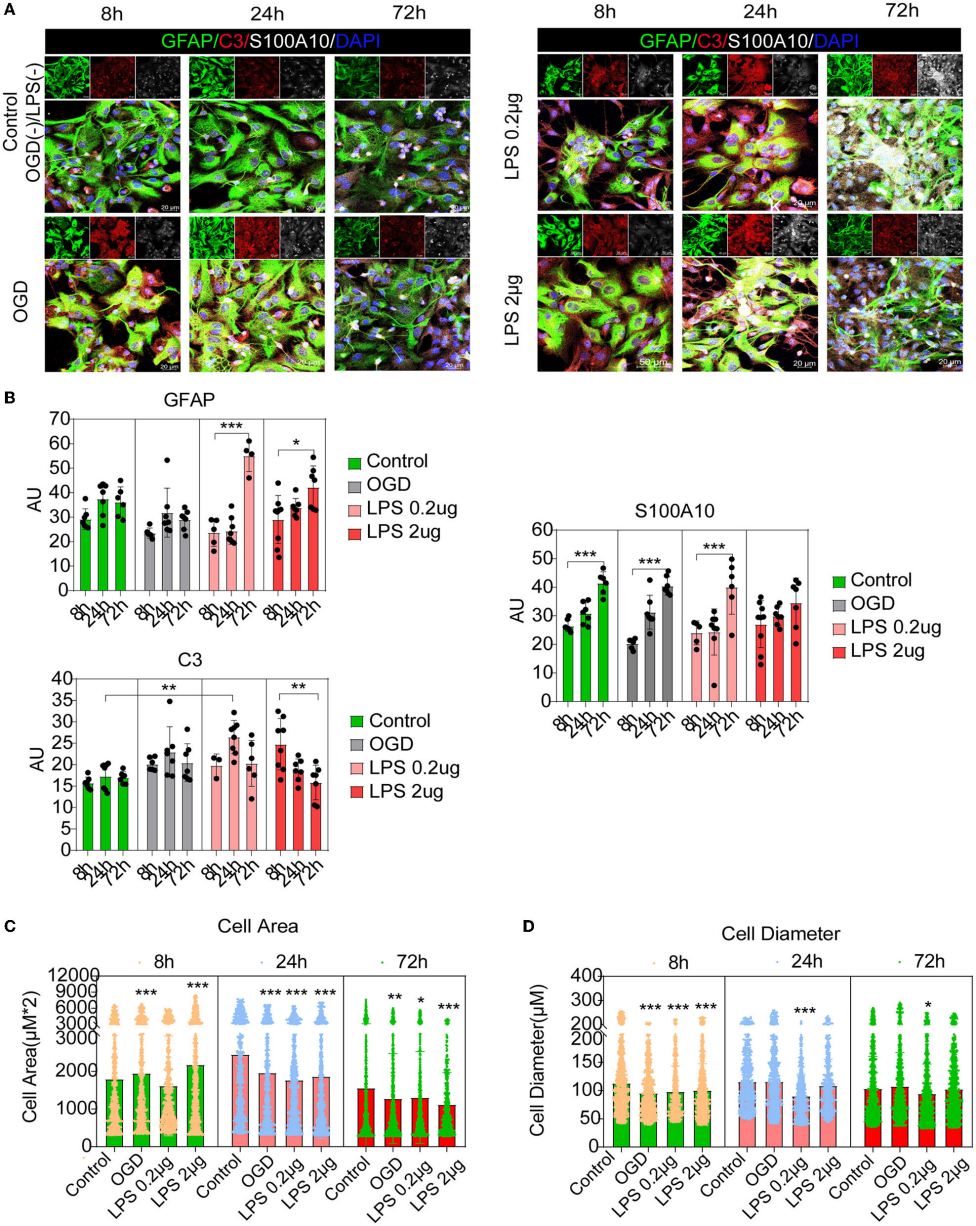
C3- and S100A10-positive reactive astrocytes underwent a dynamic transformation process at different time. **(A)** Immunofluorescence staining showed that C3- and S100A10-positive reactive astrocytes were activated under different stimulation conditions and had a dynamic transformation process. *n* = 3 biological repeats. Scale bars are indicated in the pictures. **(B)** Histogram of fluorescence intensity statistical results. *n* = 3 biological repeats. Values are the mean ± SEM. Statistical significance was determined by one-way ANOVA followed by Student Newman–Keuls *post-hoc* test. *P < 0.05, **P < 0.01, ***P < 0.001. **(C, D)** Histogram of cell area and diameter statistical results. *n* = 3 biological repeats. Values are the mean ± SEM. Statistical significance was determined by one-way ANOVA followed by Student Newman–Keuls *post-hoc* test. ***P < 0.001. LPS, lipopolysaccharide; OGD, oxygen glucose deprivation.

Recent studies have shown that C3-positive reactive astrocytes can transform into S100A10-positive reactive astrocytes (Li T. et al., 2020; Wang et al., 2021; Liu et al., 2022). Furthermore, we tried to detect the dynamic changes and transformation process under inflammatory and hypoxic conditions. A previous study reported that neuroinflammation induced C3-positive reactive astrocytes and ischemic stroke activated S100A10-positive reactive astrocytes (Zamanian et al., 2012). Thus, microglial conditioned medium (MCM) from LPS-treated microglia (mimicking neuroinflammation) and OGD experiments (mimicking hypoxic conditions) were used to stimulate astrocytes (the experimental flow chart had shown in Figure 1). Immunofluorescence staining and fluorescence intensity analysis showed that C3- and S100A10-positive reactive astrocytes were activated under different stimulation conditions and had a dynamic transformation process (Figures 5A, B). LPS stimulation could gradually activate astrocytes but not OGD stimulation and control group (Figures 5A, B). C3-positive reactive astrocytes were significantly activated after stimulation with OGD, 0.2 and 2 μg/mL LPS, and gradually decreased at 72 h (Figures 5A, B). In contrast, S100A10-positive reactive astrocytes were gradually increased in control group without OGD or LPS stimulation (Figures 5A, B). Likely, after OGD and LPS stimulation, S100A10-positive reactive astrocytes were gradually increased (Figures 5A, B). In particular, a majority of GFAP+ C3+ S100A10+ triple-positive cells were found after OGD and LPS stimulation (Figure 5A) as *in vivo* (Figure 2A), which may be the intermediate state of C3- and S100A10-positive reactive astrocytes during transformation. These results indicated that C3-positive reactive astrocytes may gradually transform into S100A10-positive reactive astrocytes after OGD or LPS stimulation. Previous studies have reported that reactive astrocytes undergo morphological changes after SCI (Wanner et al., 2013; Sofroniew, 2015) and that C3-positive reactive astrocytes show a more complex pattern of arborization (Clark et al., 2019). We also found that the astrocytes showed elongation of cell processes and overlapping cell morphology (Figures 5C, D).

**Figure 6:**
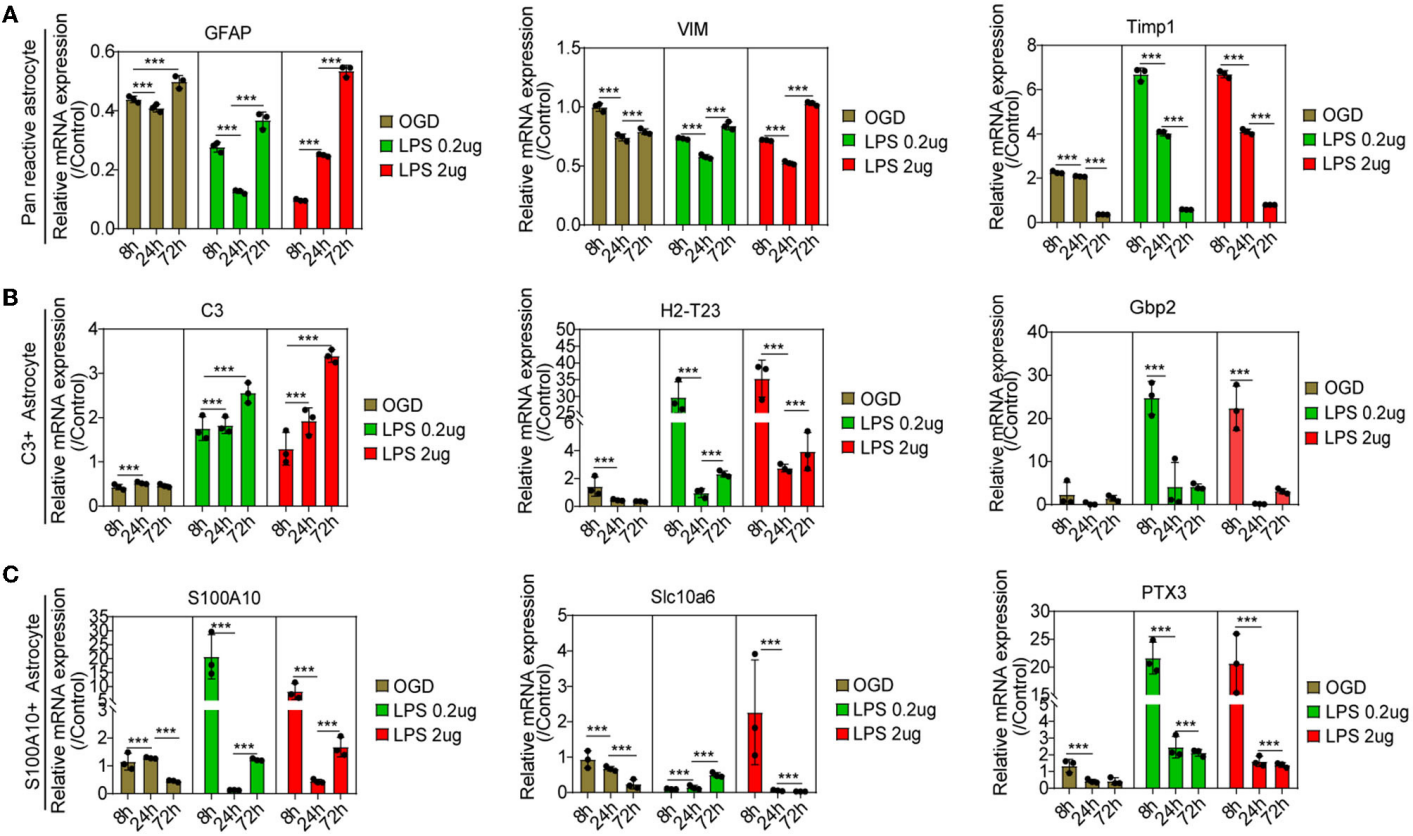
**(A–C)** RT-PCR was used to detect the mRNA expression of C3- and S100A10-positive reactive astrocytes marker genes at different time points. *n* = 3 biological repeats. Values are the mean ± SEM. Statistical significance was determined by one-way ANOVA followed by Student Newman–Keuls *post-hoc* test. ***P < 0.001. LPS, lipopolysaccharide; OGD, oxygen glucose deprivation.

Compared with the control group, astrocytes were activated at different times, as reflected by pan-reactive astrocyte markers including the mRNA expression of *GFAP, VIM*, and *TIMP1* (Figure 6A). The C3-positive reactive astrocytes were gradually activated, as shown by the mRNA expression of the C3-positive reactive astrocytes markers after OGD and MCM stimulation (Figure 6B), including the mRNA expression of *C3, H2-T23*, and *Serping1*. In contrast, the mRNA expression of S100A10-positive reactive astrocytes, including the mRNA expression of *S100A10, Slc10a6* and *PTX3*, was activated early at 8 h and then gradually decreased after OGD and MCM stimulation (Figure 6C). While, OGD stimulation did not significantly activate S100A10-positive reactive astrocytes compared with MCM intervention (Figures 6A, B). Taken together, these results indicated that C3- and S100A10-positive reactive astrocytes were significantly activated and underwent a dynamic transformation process after OGD and microglial conditioned medium stimulation.

## 4 Discussion

There were several findings in this study: (1) C3- and S100A10-positive reactive astrocytes had different cell origins from resident astrocytes and ependymal cells; (2) C3- and S100A10-positive reactive astrocytes were predominantly activated in adult and juvenile mice, respectively; (3) C3- and S100A10-positive reactive astrocytes had a dynamic transformation process at different time points *in vitro* and *in vivo;* (4) C3-positive reactive astrocytes-associated lipid toxicity-related genes were gradually increased after SCI.

One of our findings is that C3- and S100A10-positive reactive astrocytes had different cell origins from resident astrocytes and ependymal cells. Recent studies have confirmed that there are three lineages of astrocytes after SCI: resident astrocytes, ependymal cells and NG2 progenitors (Sofroniew, 2009; Hansen and Malcangio, 2013). Resident proliferation-derived astrocytes are the most abundant and mainly located around the scar, which forms a region complementary to cells derived from ependymal cells (Barnabé-Heider et al., 2010; Sabelström et al., 2014). Limiting secondary injury caused by inflammation may be a specific function of resident astrocytes, while ependymal cell-derived astrocytes may strengthen the damaged spinal cord (Sabelström et al., 2013). Ependyma is the main source of endogenous neural stem cells in the spinal cord, which widely migrate to the injury area and generate the most neuroprotective astrocytes (Meletis et al., 2008; Barnabé-Heider et al., 2010; Sabelström et al., 2013). The functions of NG2 cells, also known as oligodendrocyte progenitor cells, are still controversial (Nishiyama et al., 2005; Huang et al., 2020), and these cells are not the main source of reactive astrocytes after cortical contusion (Komitova et al., 2011; Hackett and Lee, 2016). Thus, these findings may be one of the reasons for neuroprotective S100A10-positive reactive astrocytes are mainly derived from ependymal cells.

Astrocyte heterogeneity is closely associated with neural repair and the prognosis of SCI. Astrocyte phenotypes with regard to proliferation, morphology and chemistry are heterogeneous adjacent to the crush center after SCI and change with the distance from the lesion area (Wanner et al., 2013; Khakh and Sofroniew, 2015). Furthermore, astrocyte heterogeneity also depends on the developmental stages (Pekny and Pekna, 2014). We found that astrocyte activation and glial scar formation were not evident in the juvenile mice at 1- and 2-week-old. A previous study found that postnatal day 2 mice show efficient repair of neuronal axons 2 weeks after SCI, whereas little or no axonal regeneration occurred in older mice, including postnatal day 7, day 20, and adult mice (Li Y. et al., 2020). However, we found a significant advantage of the 1- and 2-week-old mice in damage repair after SCI compared to the adult mice, which indicated that the juvenile mice < 2 weeks of age may still be capable of robust nerve repair. Importantly, we also found that C3-positive reactive astrocytes and microglia were significantly activated in the adult mice, while S100A10-positive reactive astrocytes activation was mainly increased in the juvenile mice after SCI, which may be the reason for the worse prognosis and repair capacity of the adult mice than the juvenile mice after SCI.

Increasing studies have confirmed that astrocytes have dual functions of promoting and inhibiting axon regeneration after SCI. Glial scars mainly formed by astrocytes are widely regarded as a physical and chemical barrier for axon regeneration. However, astrocytes can exert neuroprotective functions by releasing various trophic factors, such as glial cell-derived neurotrophic factor (GDNF) (Cunningham and Su, 2002) and brain-derived neurotrophic factor (BDNF) (Degos et al., 2013). Even eliminating astrocytes in the injury area and preventing glial scar formation did not promote axon regeneration (Anderson et al., 2016). Furthermore, astrocytes are heterogeneous in different injury periods. A previous study showed that growth factor expression was significantly higher in glial scars at 2 weeks than at 8 weeks (Li et al., 2018). Glial scars expressing pro-regenerative inhibin subunit β A (Inhba) and the glutamate excitotoxicity inhibitor EphA4 were gradually decreased, whereas lipotoxic Apoe/Apoj/Elovl1 and neurotoxic transglutaminase 2 (TG2) were gradually increased after SCI (Gong et al., 2022). This evidence indicated that the glial scar microenvironment gradually transformed to inhibit regeneration with scar maturation, and astrocyte function gradually changed from promoting to inhibiting axonal regeneration (Gong et al., 2022). Our results found that S100A10-positive reactive astrocytes were significantly activated on the first day and then gradually decreased, whereas C3-positive reactive astrocytes were gradually activated after SCI. Thus, the dynamic changes in neurotoxic C3- and neuroprotective S100A10-positive reactive astrocytes may be one of the reasons.

In summary, our results revealed the origins and dynamic changes in C3- and S100A10-positive reactive astrocytes after SCI, which would be valuable resources to further target C3- and S100A10-positive reactive astrocytes and for therapeutic research after SCI. Thus, selectively blocking C3-positive reactive astrocytes activation or promoting the transformation of C3-positive reactive astrocytes into S100A10-positive reactive astrocytes and naïve astrocytes may be a potential therapeutic strategy to promote nerve repair and axon regeneration in SCI. There were limitations that a majority of the cells were not distinct C3- or S100A10-positive reactive astrocytes, which due to the lack of more precise markers and transgenic mice. Meanwhile, it should be noted that the definitions of C3- and A2 phenotypes maybe misinterpreted, mainly for the functions of these genes to definite C3- and A2 or pan-reactive astrocytes have not yet verified in depth (Escartin et al., 2021). Thus, in future work, a strict lineage tracing method and more accurate markers are needed to explore their transformation and could enable us to conditionally eliminate C3- and S100A10-positive reactive astrocytes, which may further provide a potential treatment strategy for nerve repair after SCI.

## Data availability statement

The datasets presented in this study can be found in online repositories. The names of the repository/repositories and accession number(s) can be found in the article/Supplementary material.

## Ethics statement

The animal study was approved by the Institutional Animal Care and Use Committee (IACUC) of the Tongji University School of Medicine. The study was conducted in accordance with the local legislation and institutional requirements.

## Author contributions

QZ: Conceptualization, Data curation, Formal analysis, Investigation, Methodology, Software, Writing—original draft, Writing—review & editing. Y-lR: Data curation, Investigation, Methodology, Validation, Writing—original draft, Writing—review & editing. Y-jZ: Data curation, Writing—original draft, Writing—review & editing. R-qH: Investigation, Validation, Writing—review & editing. R-rZ: Conceptualization, Funding acquisition, Project administration, Supervision, Writing—review & editing. L-mC: Conceptualization, Funding acquisition, Methodology, Supervision, Writing—review & editing. NX: Conceptualization, Funding acquisition, Project administration, Supervision, Visualization, Writing—review & editing.
