# Supplementary material for: The origins and dynamic changes of C3- and S100A10-positive reactive astrocytes after spinal cord injury
Source: Front Cell Neurosci. 2023 Dec 22;17:1276506. doi: 10.3389/fncel.2023.1276506 (PMC10766709; doi:10.3389/fncel.2023.1276506)
Supplement: Supplementary file 1 [file Data_Sheet_1.PDF]

Supplementary Material

1 Supplementary Tables

Supplementary Table1.Experimental models: organisms/strains

Mouse	Cat#	Resource	Genotyping primers (5'to3')	
Mouse: *Foxj1*$^{em1(GFP\text{-}CreERT2\text{-}polyA)Smoc}$	NM-KI-200133	Model Organisms Centre, Shanghai	F1	GTTTGGGCCTTCCTACCCTC
			R1	TTCGAGATGTGCACGACGAT
			F2	TCTTTCCTCTCGGGGTAGGG
			R2	CTTGTAGTTGCCGTCGTCCT
Mouse: Ai9(tdTomato) - B6.Cg-*Gt(ROSA) 26Sor* $^{tm9(CAG\text{-}tdTomato)\ Hze}$/J	JAX:007909	The Jackson Laboratory (JAX)	F1	AAGGGAGCTGCAGTG GAGTA
			R1	CCGAAAATCTGTGGGAAGTC
			F2	GGCATTAAAGCAGCGTATCC
			R2	CTGTTCCTGTACGGCATGG

Supplementary Table 2. primers for RT-PCR

Primer name	Primers (5'to3')	
Rat-GFAP	forward	*5'-AACCGCATCACCATTCCTGT-3'*
	reverse	*5'-TCCTTAATGACCTCGCCATCC-3'*
Rat-Vim	forward	*5'-GAGGAGATGAGGGAGTTGCG-3'*
	reverse	*5'-CTGCAATTTTTCTCGCAGCC-3'*
Rat-Timp1	forward	*5'-CGCTAGAGCAGATACCACGA-3'*
	reverse	*5'-CCAGGTCCGAGTTGCAGAAA-3'*
Rat-C3	forward	*5'-GCTCATCAAGAAAGGGTACA-3'*
	reverse	*5'-CCACCTCCGTAGTATCTTTG-3'*
Rat-H2-T23	forward	*5'-AGAAGTCCACCATTCCTCTT-3'*
	reverse	*5'-TAGTTACAGACAGCGAGTTA-3'*
Rat-Serping1	forward	*5'-AGGCTAACTGGCTTCGTA-3'*
	reverse	*5'-CGTGGTAGAGTTTCACAGAG-3'*
Rat-S100A10	forward	*5'-ATTCTTCAAAATGCCATCCC-3'*
	reverse	*5'-GACTTTCTTTATTGAGGGCA-3'*
Rat-Slc10a6	forward	*5'-GTCTGGTCATGTTCTCCTTT-3'*
	reverse	*5'-GTCTCTATGGAAATCGTCCT-3'*
Rat-Ptx3	forward	*5'-TGCATGTGAATTTGGACAAC-3'*
	reverse	*5'-TTCATTGGTCTCACAGGATG-3'*
